# Hominin and animal activities in the microstratigraphic record from Denisova Cave (Altai Mountains, Russia)

**DOI:** 10.1038/s41598-019-49930-3

**Published:** 2019-09-26

**Authors:** Mike W. Morley, Paul Goldberg, Vladimir A. Uliyanov, Maxim B. Kozlikin, Michael V. Shunkov, Anatoly P. Derevianko, Zenobia Jacobs, Richard G. Roberts

**Affiliations:** 1 Centre for Archaeological Science, School of Earth, Atmospheric and Life Sciences, University of Wollongong, Wollongong, New South Wales 2522 Australia; 2 Archaeology, College of Humanities and Social Sciences, Flinders University, Adelaide, South Australia 5042 Australia; 3 Institut für Naturwissenschaftliche Archäologie, Eberhard-Karls-Universität Tübingen, Rümelinstrasse 23, Tübingen 72070 Germany; 4 Institute of Archaeology and Ethnography, Russian Academy of Sciences, Siberian Branch, Novosibirsk 630090 Russia; 5 Lomonosov Moscow State University, Moscow, 119991 Russia; 6 Novosibirsk State University, Novosibirsk, 630090 Russia; 7 Australian Research Council (ARC) Centre of Excellence for Australian Biodiversity and Heritage, University of Wollongong, Wollongong, New South Wales 2522 Australia

**Keywords:** Evolution, Environmental social sciences

## Abstract

Denisova Cave in southern Siberia uniquely contains evidence of occupation by a recently discovered group of archaic hominins, the Denisovans, starting from the middle of the Middle Pleistocene. Artefacts, ancient DNA and a range of animal and plant remains have been recovered from the sedimentary deposits, along with a few fragmentary fossils of Denisovans, Neanderthals and a first-generation Neanderthal–Denisovan offspring. The deposits also contain microscopic traces of hominin and animal activities that can provide insights into the use of the cave over the last 300,000 years. Here we report the results of a micromorphological study of intact sediment blocks collected from the Pleistocene deposits in the Main and East Chambers of Denisova Cave. The presence of charcoal attests to the use of fire by hominins, but other evidence of their activities preserved in the microstratigraphic record are few. The ubiquitous occurrence of coprolites, which we attribute primarily to hyenas, indicates that the site was visited for much of its depositional history by cave-dwelling carnivores. Microscopic traces of post-depositional diagenesis, bioturbation and incipient cryoturbation are observed in only a few regions of the deposit examined here. Micromorphology can help identify areas of sedimentary deposit that are most conducive to ancient DNA preservation and could be usefully integrated with DNA analyses of sediments at archaeological sites to illuminate features of their human and environmental history that are invisible to the naked eye.

## Introduction

**Figure 1:**
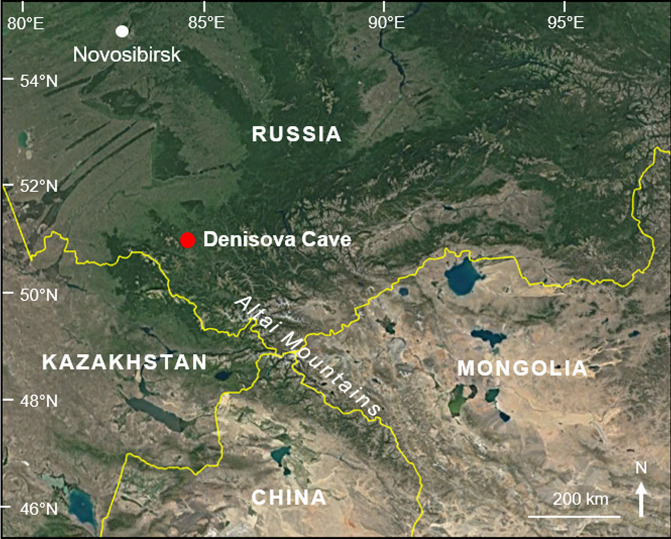
Location of Denisova Cave (red dot) in the foothills of the Altai Mountains in southern Siberia (Satellite imagery: GoogleEarth, DigitalGlobe).

Situated in the foothills of the Altai Mountains in southern Siberia, Denisova Cave (51°23′51″N, 84°40′36″E; Fig. 1) occupies a prominent place in world prehistory due to the hominin fossils and ancient DNA (aDNA) recovered from the site and the sediments preserved within. These finds have revealed two Pleistocene archaic human populations: a hitherto unknown hominin group, the Denisovans, identified chiefly through genome sequencing of fossils; and Neanderthals, who are at the eastern limits of their known range at this site^1–3^. The hominin story that has emerged from this cave since publication of the Denisovan genome has stimulated considerable scientific debate and media attention. Much of this deliberation has centred on the role of the Denisovans in the recent evolution of our own species and, more broadly, to the demographic composition of Late Pleistocene Eurasia^4,5^.

Archaeological research has been ongoing at Denisova Cave for four decades, during which a great deal of data has been generated regarding the nature and timing of occupational pulses, and the environmental and ecological context of site use^6–9^. The cave is situated above the right bank of the Anui River at a point where the valley narrows. The present-day opening of the cave is ~30 m above the modern-day river level, penetrating into the Silurian limestone bedrock along a series of interconnected karstic chambers and tubes that are currently filled with both natural and cultural sediments.

Recent excavations of the three principal chambers (Main, East and South Chambers) continue to demonstrate the complexity and spatial heterogeneity of the deposits, revealing a stratigraphic sequence created by geological (e.g., physical and chemical weathering, subsidence and deformation), biological (e.g., animal activity) and anthropogenic (e.g., stone artefact manufacture) processes^9^. Although the cave remains in the spotlight because of the hominin fossils and aDNA found at the site^10–12^, these items account for only a small fraction of the total material recovered. Much additional information about their immediate and regional context can be gleaned from the study of the deposits that encase them and the cultural remains.

A new program of geoarchaeological investigation was initiated in 2014, aimed at documenting the microstratigraphic aspects of the depositional and post-depositional environments represented in the deposits. This work employs micromorphology and contextually-specific elemental mapping of the sediments to seek trace-evidence of processes that formed the site and obtain insights into the behaviours and activities of the site’s occupants—both hominins and other animals—and contextualise at high resolution the artefacts, fossils and genetic material.

In particular, our purpose is to: (i) investigate micro-scale evidence for occupation of the cave by hominins and other animals that is not visible at the field scale (e.g., microscopic traces of fire-use, trampling, denning and middening); (ii) further refine our understanding of the genesis of the deposits, including the naturally occurring (geogenic) inputs and post-depositional modifications, and potential correlation to micro-environments in the cave; and (iii) provide an enhanced sedimentary context for the artefacts and for the fossils and sediments from which aDNA has been extracted^1–3,9–12^. The latter is of special interest given the high degree of aDNA preservation at Denisova Cave.

Here, we report the micromorphological results for nine undisturbed sediment blocks collected in 2014 from the Main and East Chambers (DCM and DCE, respectively). Micromorphological analyses retain micro-scale physical relationships^13^, whereas other analytical techniques commonly employed in archaeological investigation (e.g., zooarchaeological, palynological and stone tool analyses) require the removal of material from its original context (i.e., from the sedimentary matrix) before analysis, resulting in the loss of critical contextual information and associations with complementary datasets.

The deposits considered here partly fill DCM and DCE, solution cavities that were formed early in the cave’s history under phreatic and hydrothermal conditions^6^, most likely at a time well before exposure of the cave to the sub-aerial environment during and after downcutting of the Anui River^14^. The sediments in DCM and DCE have been described previously from field observations and sedimentological analyses^6,7,9,14,15^, with the earliest stone artefacts recovered from sediments deposited ~300 thousand years ago (ka)^9^.

**Figure 2:**
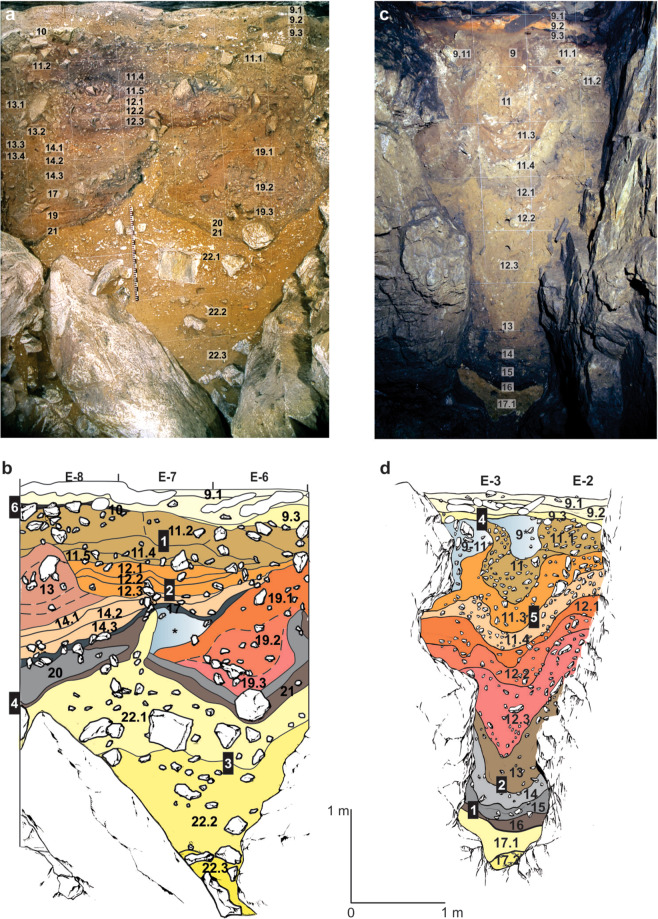
Stratigraphic sequences exposed in the Main (DCM) and East (DCE) Chambers of Denisova Cave. (**a**) Southeast profile in DCM after excavations in 1984 and (**b)**, Locations of sediment block samples collected in 2014. The concavities on either side of deformed lighter yellow layer 22 (which contains the lowermost artefacts) are infilled with sediments (layers 21–14) that are, in turn, overlain by sub-horizontal layers 13 to 9. (**c**) Southeast profile in DCE after excavations in 2013 and (**d**) Locations of sediment block samples collected in 2014. The Pleistocene sequence of hominin occupation spans layers 15 to 9. Layers 17–11 sag towards the centre, above the choke point, and are overlain by sub-horizontal layer 9. Black boxes in panels (**b**,**d**) indicate the positions of the micromorphology samples, and the corresponding sample numbers are inset in white; each sample produced 2–3 thin sections, labelled alphabetically from top to bottom. DCM micromorphology samples 4 and 6 were collected from the adjacent east profile.

The sediments exposed in DCM (Fig. 2a,b) consist largely of fine-grained silts and clays containing angular limestone clasts, with occasional beds dominated by coarser limestone gravel. The lithological layers commonly exhibit abrupt bounding surfaces that are horizontal towards the upper part of the profile, whereas in the lower parts of the sequence they tend to be contorted and convoluted. The lowest unit in DCM (layer 22) has been deformed and subsequently planed by low-energy humid colluviation as seeping water entrained fine-grained sediments producing localised gullying. This process was initiated by slumping, subsidence and plastic deformation, forming two concave depressions, and it is this topographic template that has influenced much of the earlier phases of subsequent sediment deposition. Figure 2b shows the locations of the five micromorphological samples analysed from DCM.

The sediments preserved in DCE (Fig. 2c,d) are more fine-grained than those in DCM, with many layers significantly enriched with clay. DCE comprises a lower, remnant phreatic tube filled with archaeologically sterile silts and clays, and an upper chamber filled with sediments that contain evidence of both hominins and other animals; the two parts are connected by a narrow slot or choke point. The interfaces between adjacent lithological layers in DCE are convolute, with the sediments centred above the phreatic tube showing downward slumping towards this feature. Figure 2d shows the locations of the four micromorphological samples analysed from DCE.

## Results

### Hominin activity in the Denisova Cave microstratigraphic record

Hominin fossils and aDNA have been recovered from the sediments preserved at Denisova Cave^1–3,9–12^, as well as significant numbers of stone artefacts and faunal remains, specimens of which show signs of human modification^6–9,12,16,17^. Optical ages^9^ indicate slow rates of net sedimentation, with periods of non-deposition or erosion, resulting in the accumulation of up to ~4.5 m of Pleistocene deposit in DCM and DCE since ~300 ka (excluding unconformities). Unequivocal signs of hominin activity in the sediments at the field scale are limited, including evidence for fire-use^18–23^ in the Middle Palaeolithic deposits that form the majority of the sequence.

We sought microscopic evidence of hominin activity in the sediments, where diagnostic features invisible to the naked eye might be recognised. While we do not identify intact combustion features—common elements of Palaeolithic cave sites—we do observe disassembled combustion bi-products, including micro-charcoal, charcoal fragments and occasional localised ashes. Micromorphological descriptions of all samples examined in this study are provided in Supplementary Information (Table S1), together with a selection of photomicrographs of the thin sections (Fig. S1) and flatbed scans of the sediment blocks (Fig. S2).

**Figure 3:**
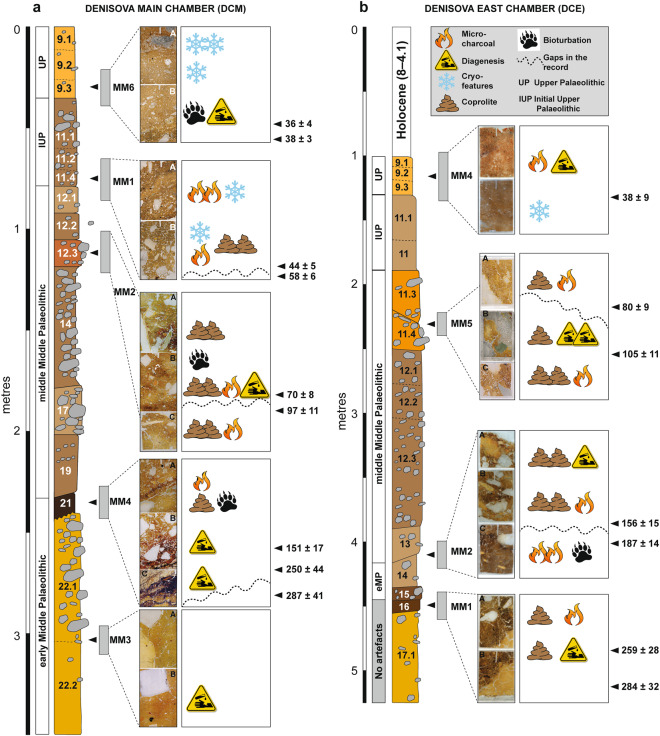
Summary stratigraphic logs of the sequences exposed in (**a**) DCM and (**b**) DCE, showing the locations of the micromorphological samples and key microstratigraphic features. To the right of each log, optical ages (in ka, with uncertainties at 95.4% probability) are shown for the major boundaries between lithological units in the thin sections, together with the associated archaeological phases (from ref.^9^).

In DCM (Fig. 3a), we observe micro-charcoal in the basal region within layer 20 (which contains early Middle Palaeolithic artefacts and finished accumulating 170 ± 19 ka), at the interface with overlying layer 19, and as a distinct band within layer 19 (which contains middle Middle Palaeolithic artefacts and started accumulating 151 ± 17 ka). (Archaeological phases and ages, with uncertainties expressed at the 95.4% confidence interval, are from ref.^9^ (Fig. 3 and Extended Data Table 1) and shown here in Fig. 3 and Table S1.) This micro-charcoal is most likely a taphonomic concentration of combustion bi-products, given the undulating topography of this part of the cave produced by deformation, the truncation of layer 22 by low-energy colluviation, and the localised concentrations of fine charcoal. Layer 19 has produced a total of 1,925 stone artefacts^9^, so clearly hominins were present at this time. Given the slow sedimentation rate, the artefact assemblage may perhaps represent the product of periodic visitations over many millennia. Site conditions would not have been attractive for hominin occupation during the deposition of these lower layers, owing to the irregular surfaces and occasionally humid conditions in the cave. Higher up the DCM sequence, layers 11.4 and 11.2 also contain charcoal, with larger fragments recorded in layer 11.4 and much finer charcoal powder in layer 11.2. Both layers are associated with the Initial Upper Palaeolithic^9^.

In DCE (Fig. 3b), we record trace quantities of fine charcoal fragments and flecks closely associated with crushed charcoal and bone fragments in layer 16, and more commonly in layer 15—the earliest layer containing artefacts (early Middle Palaeolithic)^9^ and also Denisovan DNA^10^. This indicates fire use and trampling occurring tentatively from 259 ± 28 ka and certainly from 203 ± 14 ka, but we cannot unequivocally and directly link the manufacturers of the stone tools with fire-use because these fine combustion products are highly mobile. In layer 14, which yielded Neanderthal DNA and early Middle Palaeolithic artefacts and was deposited between 193 ± 12 and 187 ± 14 ka, there is a marked increase in micro-charcoal that imparts a dark colouration, and also a small piece (3–4 mm) of angular chert debitage in our sample.

We identify large charcoal fragments (>4–5 mm) in layers 11.4 and 11.3 in DCE, which were deposited between 120 ± 11 and 70 ± 8 ka and contain middle Middle Palaeolithic artefacts. A Neanderthal toe phalanx (Denisova 5) was recovered from layer 11.4, but we cannot confidently associate this and other similarly small and isolated hominin fossils with elements of the sedimentary matrix, given the possibility of displacement^9,12^. In thin section, we observe sediment movement in the form of micro-faulting and slippage features in these layers, presumably associated with the aforementioned post-depositional subsidence. However, such deformation processes do not necessarily promote the translocation or mixing of fine material across lithological boundaries. Our sample location was close to the rear of DCE, where the chamber tapers to a narrow slot (~1 m wide), a locale unlikely to have been conducive to human occupation—especially the lighting of a fire—given the confined space. Evidence of sediment compaction does suggest compressive forces, however, so animals that are represented in the faunal record may have been present in this restricted space.

Ashes are present in very low quantities in the Denisova Cave microstratigraphy. Nevertheless, we cannot rule out ash dissolution as the biasing factor, given the mobility of calcite and decalcification recorded locally in some layers. Could fire have been used at Denisova Cave more extensively by hominins, but with the associated evidence subsequently removed from the stratigraphic record? Bearing in mind the rate of cave sedimentation, erosional (chronological) gaps and the evidence for bioturbation in some parts of the sequence—mostly parts of the upper layers of DCE—reworking and redistribution of combustion bi-products may have occurred, although it is unlikely that all micro-traces would have been completely removed. The reworking of previously *in situ* fire residues is supported by the absence of structured combustion features that would signify the presence of intact hearths. Furthermore, stone tools do not exhibit signs of thermal alteration^24^, which might be expected should fire-use have been common—or even present—in these confined spaces, and other indicators of fire, such as thermally altered clays, were not evident in our samples. Although fire may not have been used extensively within the sampled areas of the cave, the lack of an obvious pyrotechnology need not preclude the use of a site by hominins, even during glacial periods^25^. Elsewhere in the Altai, the site of Kara-Bom contains well-preserved hearths in the Initial Upper Palaeolithic deposits^26^, but no clear evidence of fire-use has been found in the region beyond about 50–40 ka^27^.

Overall, the microstratigraphic record for Denisova Cave indicates that human activity was intermittent over the past three glacial–interglacial cycles represented by the Pleistocene sedimentary infill (>300 ka to ~20 ka). The stone artefact assemblages indicate long-term hominin occupation of the site during both warm climates and cold conditions, when the foothills of the Altai Mountains likely served as a refugium^28^.

### Other animal users of Denisova Cave: the fossil coprolites

**Figure 4:**
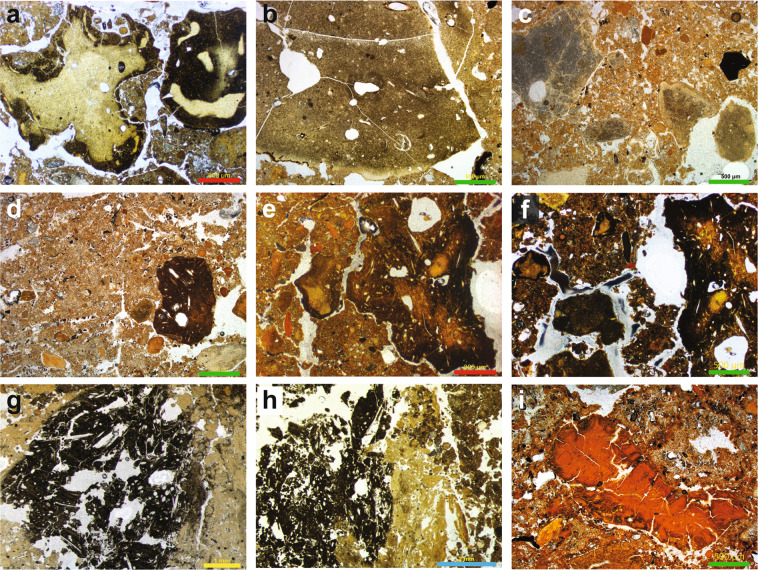
Examples of coprolites identified in the Denisova Cave microstratigraphic record (see Table 1 for coprolite descriptions). (**a**–**c**) Type CT-1 originating from cave hyena occurs through much of the sampled sequence; (**d**–**f**) Type CT-2 has a much darker matrix, possibly related to wolf; (**g**,**h**) Type CT-3 is highly weathered; (**i)** Type CT-4 has a distinctive red matrix. Types CT-3 and CT-4 cannot be linked to specific animals. Scale bars: red, 800 µm; green, 500 µm; yellow, 1 mm; blue, 2 mm.

Coprolites are common biogenic components of the cave sediments, often present in dense concentrations, suggesting that animals visited the site for much of its depositional history. The coprolites can be grouped into a number of recurring types throughout the sequence, presumably reflecting the use of the site by a variety of animals, and potentially associated with a range of preservation states. We recognise four main coprolite types (CT-1 to CT-4), described in Table 1 and shown in Fig. 4. Although we cannot confidently attribute all of these droppings to a specific animal, we assign CT-1 to *Crocuta crocuta spelaens* (cave hyena), based on consistency with published results describing the morphology and optical properties of this material in thin section^29–31^; this supports the faunal evidence of regular use of the cave by these animals^6–9^. We tentatively attribute CT-2 to wolf (*Canis lupus*), based on the similarity between these coprolite fragments and dog coprolites recorded at Vanguard Cave, Gibraltar^32^, as well as other published data^31^. The coprolite fragments (CT-3) in our thin section of layer 12.2 in DCM are consistently larger and darker than CT-1 and CT-2. This layer contains very high proportions of these coprolites, and the chaotic arrangement of the coarse limestone gravel, with long axes in a vertical to sub-vertical alignment, suggests disturbance of these sediments, possibly by a large animal such as a cave bear. We cannot assign CT-3 or CT-4 to a specific species.

**Table 1:** Coprolite types identified in the Denisova Cave microstratigraphic record.

Coprolite type	Description^a^	Occurrence (Chamber, layer)
CT-1	Rounded to sub-angular, with a pale yellow and homogeneous fabric (ppl), a ‘dusty’ composition, and a darker brown rim. The undifferentiated b-fabric is isotropic in xpl. Inclusions of bone and hair, and vesicles (void spaces), in these coprolites are generally small, although some contain larger (~1 mm) bone fragments. These are the most common coprolites recorded at Denisova Cave, most likely formed by hyenas based on published descriptions of this material^29–31^.	DCM 21, 20, 19, 14.1, 12.3, 11.4, 11.2, 10, 9.3 DCE 16, 14, 11.4, 11.3
CT-2	Rounded to sub-rounded, medium to dark brown (ppl) with frequent inclusions, such as fine sand and silt grains, and hair/fur (DCM-MM2A/B; DCE-MM2B), which are isotropic in xpl. This category is the next most common recorded in the microstratigraphy and are distinctive for their very dark colouration, similar to dog coprolites found at Vanguard’s Cave, Gibraltar^32^. They may correlate with wolf, the remains of which have been found at Denisova Cave.	DCM 14.1, 12.3, 12.2 DCE 14, 13
CT-3	Highly weathered, dark brownish black, with frequent sand-sized grains and/or vesicles, recorded in DCE-MM2A. These are highly fragmentary and their origin is unknown.	DCE 14, 13
CT-4	Moderately weathered and cracked, bright brownish red, sub-rounded, recorded in DCM-MM2B. These have a darker brown rim, and the fabric is largely homogeneous and free of inclusions, except for some fine silt grains.	DCM 14.1, 12.3

^a^ppl, plane-polarised light; xpl, cross-polarised light.

Coprolite fragments commonly occur in layers that also contain stone artefacts. Given that hominins and hyenas will not cohabitate^7,33^, this indicates that specific occupation events will be extremely challenging—if not impossible—to tease out at Denisova Cave, with the slow sedimentation rates effectively precluding the identification of alternating hominin–carnivore occupations, should they exist. The lack of defined stratigraphy within layers (e.g., buried surfaces) may be due, at least in part, to carnivore denning or other animal burrowing activities in parts of the deposit. At Bois Roche in France, for example, stone tools accumulated by local movement (e.g., by gravity) in areas that functioned primarily as carnivore dens^34^. The DCE faunal record includes the remains of a number of large cave-dwelling mammals recovered in relatively high numbers, including hyena, wolf, red fox and, to a lesser extent, bear^9,35^. As these animals are unlikely to have cohabitated, either with each other or with hominins, the co-occurrence of their remains likely reflects the scale of resolution (time averaging) of the sedimentological, chronological and hominin occupation records at Denisova Cave.

The presence of coprolites in layers from which hominin remains and aDNA have been recovered implies that large carnivores might be an accumulating agent for these materials, particularly in areas of the site where evidence for hominin activity is scarce (e.g., the farthest recesses of DCE). Specific areas of the site might have been designated as waste dumps for lithic debitage and food detritus, for example, which in turn attracted scavengers such as the cave hyena when hominins were absent from the site. Interestingly, we record in thin section only a few examples of bone fragments that exhibit characteristic etching related to digestion in the gut of a carnivore, although etched bones are common in the faunal record^35^.

In the field, rodent burrows (krotovinas^36^) are clearly visible in the Holocene deposits of DCE, and in fewer numbers in DCM. Disturbance of the sediments by bioturbation is also evident in thin section. Parts of layer 12 and much of layer 13 in DCM display a chaotic arrangement of limestone clasts within finer material, consistent with disturbance by large animals such as bears, wolves or hyenas. This accords with field observations of layer 13 being a hyena lair^9^. Thin sections of layers 9.2 and 9.3 in DCM and layers 9.1, 11.3 and 11.4 in DCE display abundant, loosely arranged aggregates and irregular vughs typical of bioturbation^37^. These small, mm-size features are typical of smaller soil fauna, such as worms, spring-tails (Collemboles) and isopods. We note that these fine crumb structures occur essentially in the uppermost Pleistocene strata in DCE (i.e., layer 9), which accumulated after 38 ± 9 ka and may represent milder conditions that enabled these fauna to flourish.

### Diagenesis and the completeness of the archaeological record

**Figure 5:**
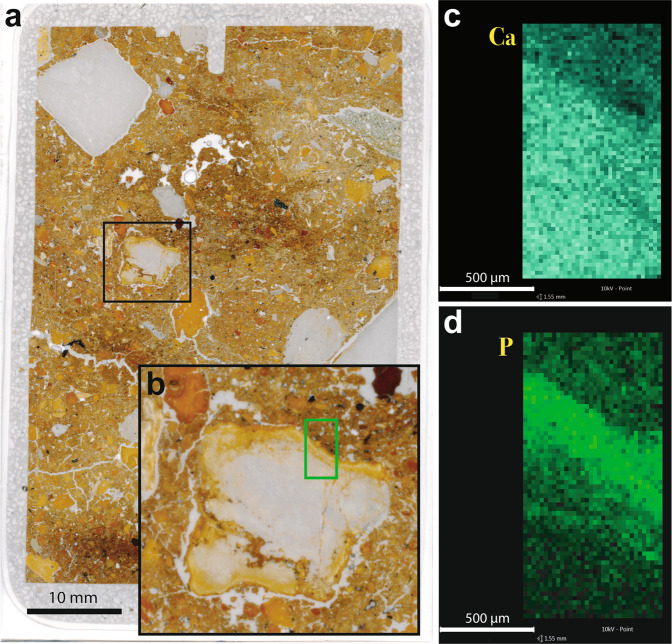
Phosphate rind around limestone grain in sample DCM-MM2B. (**a**) Macroscan of thin section of this sample; (**b**) Inset showing limestone fragment, with green rectangle indicating the location of qualitative maps collected using energy-dispersive X-ray (EDS) spectroscopy. EDS maps showing the relative distribution of (**c**) calcium and (**d**) phosphorus, in which higher colour intensities represent greater concentrations of each element.

Chemical alteration features are rare in Denisova Cave. Where present, they take the form of carbonate dissolution and phosphatisation, such as that reported for the uppermost Pleistocene and Holocene layers in DCE^38^. In thin section, we observe phosphatic rinds around limestone clasts, a common occurrence in prehistoric caves when calcite reacts to phosphate-rich solutions^39–44^. This is expressed as reaction rims around individual clasts (Fig. 5), resulting in replacement of the original birefringent calcite by isotropic phosphate, generally apatite (dahllite). We also record the etching of calcite sand and decalcification of the surrounding matrix (e.g., in layer 13 in DCE), indicating the dissolution of calcium carbonate.

Animals are the most likely source of phosphate in an archaeological cave sequence^40^. Although some layers are richer than others in phosphates, including coprolites, none of those examined in thin section stands out as being excessively phosphatised. Bat and bird guano is also a possible source of cave phosphates and associated diagenetic transformations^21,38,40–51^. We did not record guano directly in thin section, but acidic water percolating through guano—in combination with coprolite-rich sediments—can dissolve calcite^21^, including the fine calcareous fraction of limestone grains and the outer surface of larger limestone fragments, to produce apatite (dahllite) rims. Bat remains occur in relatively high numbers in some layers^9,35^. Although bats do not commonly occupy caves at the same time as hominins, small populations could, nonetheless, have supplied a persistent supply of guano to maintain phosphatisation processes.

Common diagenetic cave minerals (e.g., taranakite, leucophosphite, crandallite, brushite and ardealite)^48,52–55^ have been recorded in the Holocene deposits and in layer 11.1 in DCE^38^. The diagenesis occurred during the Holocene and affected only the upper parts of the underlying Pleistocene sediments. We do not observe these minerals in our thin sections of the Pleistocene deposits, underscoring Denisova Cave as a depositional environment where persistently cold conditions have afforded exceptional preservation of organic materials—including lipid micro-residues on Middle Palaeolithic stone tools in DCE^56^—and minimal diagenesis.

### Cold-climate indicators and implications for cave use

We record platy microstructures in thin section for layers 12.2 (70 ± 8 to 58 ± 6 ka) and 11.4/11.2 (44 ± 5 to 38 ± 3 ka) in DCM, and for layers 13 (156 ± 15 to 146 ± 11 ka) and 11.1/9.1 (49 ± 8 to after 38 ± 9 ka) in DCE. These features, together with the presence of rounded grains and granostriated b-fabrics, which are indicative of grain rotation, indicate incipient cryoturbation. This modification of the sediment structure is most likely associated with seasonal frost, with the thinner bands in layer 9.1 of DCE possibly associated with repeated ice lensing as a result of soil creep during thaw^57^. The limestone clasts in these parts of the stratigraphy are generally angular and fresh, and lack signs of phosphatisation that would reflect diagenetic transformations of calcite. We therefore correlate these platy structures with the occurrence of low temperatures in the cave and relatively few freeze-thaw cycles^58^.

**Figure 6:**
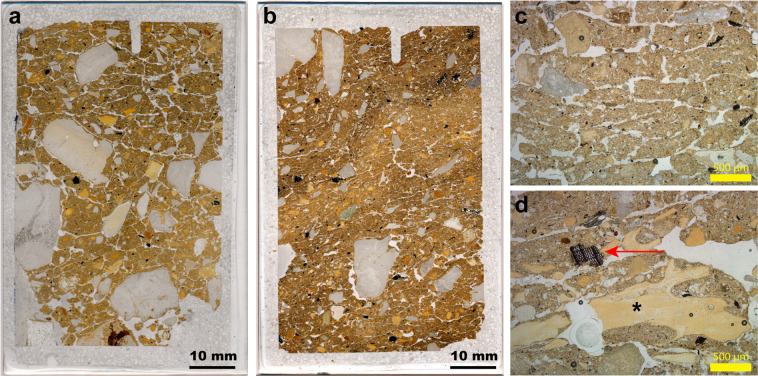
Evidence of freezing conditions in the microstratigraphy at Denisova Cave. (**a**,**b**) Two thin-section scans from layers 11.4 and 11.4/11.2 in DCM, respectively; (**c**) Photomicrograph showing detail of the platy structures relating to frost heave; (**d**) Photomicrograph showing the excellent state of preservation of bone (asterisk) and charcoal with preserved plant cellular structure (red arrow).

In DCM, these platy microstructures are associated with sediments that contain unequivocal signs of hominin occupation (charcoal and closely associated bone fragments; Fig. 6). We do not know the vertical extent of these post-depositional features, however, so it is not clear how these signatures correlate. They may penetrate down into the underlying, older layers, but the sediments immediately above and adjacent to these samples are not affected in this way. Given the slow rate of sedimentation in the cave, we cannot rule out later over-printing of the sediments by these cold-climate indicators.

At the present day, thin (mm-thick) vertical cracks filled with ice have sometimes been observed within the Holocene deposits in the South Chamber. These would not, however, account for the horizontal ice lensing observed in our thin sections of layers 11.2 and 11.4 in DCM, and we see no such analogous vertical fissures in any of our thin sections. It is not clear why such platy structures and signs of incipient cryoturbation are not more common in the Pleistocene sequences at Denisova Cave, but this may relate to the enclosed cave environment mitigating extremes in temperature through restricted airflow. The South Chamber is better ventilated than are DCM and DCE, which may explain their modern occurrence there.

## Discussion

Micromorphological analysis of the Denisova Cave sequence has provided micro-contextualised insights into the use of the site by hominins and other animals. These new data largely support previous interpretations based on field observations and other proxy datasets (e.g., faunal and pollen records^6–9,35,59^), thus increasing confidence in environmental reconstructions for the cave and surrounding region.

Microscopic evidence for hominin use of the cave is minimal. Decades of excavation have generated significant numbers of stone artefacts^6,9^ that accumulated over a substantial time interval and represent multiple occupational pulses. Micro-remains of a hominin presence—such as combustion bi-products—are readily mobilised and re-deposited, so the lack of intact features indicating fire-use in the Pleistocene sequence is intriguing. Given the limited spatial area that our micromorphological study encompasses, this outcome could be due to sampling bias (e.g., ref.^60^). On the other hand, because easily dispersed combustion bi-products are, nonetheless, very rare, the early occupants of the site may not have been prolific pyrotechnologists. Where we do record charcoal, it is usually well preserved, so we can rule out the possibility of complete degradation of this material and its preferential removal from the sedimentary record.

The abundant coprolite record shows that the cave was occupied by animals near-continuously. Cave hyena (*Crocuta crocuta speleans*)—the dominant carnivore in the Altai during the Pleistocene^6,59^—was present throughout the period of deposition of the Pleistocene sediments^7,9,35^. Whereas bones can accumulate at a cave site through the agency of various animals, animal droppings are most likely to be delivered directly to the cave floor. Coprolites, then, can be viewed as authigenic components of the sedimentary fill and we surmise that animals—mainly carnivores—used the sampled area of the cave throughout the time represented by the preserved sediments. Fossils of cave hyena are considerably more frequent in the DCE faunal record than are those of other Pleistocene predators, such as wolf, so hyenas are most likely the main accumulating agents of the faunal remains, given the dominance of their coprolites in the cave sediments.

Coprolite fragments occur in high frequencies in layers that have been affected by frost action (e.g., layers 11.4 and 11.2 in DCE). We cannot rule out the possibility that the sampled areas fall within specific latrine areas used for ‘social defecation’^61,62^, perhaps exacerbated by colder temperatures driving the animals into the further recesses of the cave. At Zhoukoudian Cave in northeastern China^60^, rich coprolite concentrations and signs of trampling were recognised from sampling localities close to the walls of the cave, confirming the attraction of these animals to marginal zones. Profiles sampled close to the walls of caves may, therefore, fall in areas favoured for animal latrines, underscoring the importance of sampling at multiple locations throughout a site.

An important outcome of our study is the identification of microstratigraphic features consistent with freezing conditions. At present, even on a sunny summer’s day, the cave interior is cold, especially so in DCE, due to the high thermal mass of the surrounding rock mitigating the warm temperatures experienced outside the cave. The cave sediments are frozen during the winter months, when temperatures can drop to an average of −16 °C in January^9^, but apparently not to the extent that the sediment fabric is re-arranged by ice lensing. The platy structures developed in layers deposited during late Marine Isotope Stage (MIS) 4 and MIS 3 in DCM, and during MIS 6 and MIS 3 in DCE, suggest colder conditions than those experienced in the current interglacial. We postulate that the platy features observed do not necessarily reflect the coldest conditions at the site during the Pleistocene, but are associated with specific formation environments—cold and humid conditions—that allow ice lensing to occur^63^.

While we observe incipient platy structures in sediments dated to terminal MIS 3 and early MIS 2 (e.g., layer 9, deposited between about 40 and 20 ka), and thus overlapping with the coldest period of the last glacial cycle, the platiness is best expressed in layers that accumulated during the preceding, comparatively warm MIS 3. There is debate surrounding the synchronicity of glacial expansion in the Altai with global climate proxies^64,65^, potentially associated with increased moisture availability in central Asia during MIS 3 relative to MIS 2, which was drier. The Denisova Cave microstratigraphic record may, therefore, document both cold and relatively humid conditions^66^, sufficient to develop platy microstructures in the near-surface, cave floor sediments.

Our study provides spatially resolved information on the depositional and post-depositional environments that have preserved organic materials in Denisova Cave. Throughout the sequence, we record only limited evidence of severe chemical diagenesis in the Pleistocene layers, but these include layers that have also yielded sedimentary aDNA^10^. Even in these layers, however, the evidence for diagenesis is generally weak, albeit spatially variable, providing opportunities for DNA preservation. Cold conditions may also promote the survival of genetic material and lipids in coprolites, from which aDNA and faecal biomarkers can be extracted directly for species identification and dietary reconstruction^67,68^.

## Conclusions

The deposits in Denisova Cave contain microscopic traces of hominin and animal activities that illuminate the use of the cave over the last three glacial–interglacial cycles (Fig. 3). The micromorphological results show that the cave sediments are predominantly geogenic (naturally occurring), augmented by biogenic (biological) additions (e.g., coprolites, guano and digested bone) and anthropogenic inputs (e.g., charcoal, stone artefacts and associated debitage).

Relationships between the various lines of evidence (e.g., micro-charcoal, bioturbation, coprolites and diagenesis), examined at a finely resolved spatial scale, reveal that hominin activities in the microstratigraphic record are few. On the other hand, coprolitic evidence for cave-dwelling carnivores is ubiquitous and suggests that the site often served as a den for hyenas and, to a lesser extent, for wolves.

The cave was visited sporadically by hominins, who appeared not to have been prolific users of fire, at least in the Middle Palaeolithic deposits that constitute the majority of the Pleistocene sequence. The low frequency of hominin occupation has implications for determining the potential agency by which the few Denisovan and Neanderthal fossils were introduced to the site and their post-depositional stratigraphic integrity^9,12^.

The environmental conditions that best preserve organic molecules, such as DNA and lipids, and whether these materials can be recovered from specific components of the microstratigraphy (e.g., coprolite fragments), also warrant further investigation. Ongoing work at Denisova Cave aims to more fully integrate micromorphology and sedimentary aDNA analyses to develop a predictive tool for organic material preservation in the deposits at this unique hominin locality.

## Methods Summary

Micromorphology is the study of intact sediment blocks, principally using petrographic thin sections^69–71^. As the original geometric arrangements within the blocks are retained, micromorphological observations allow the original relationships to be observed. Other microanalytical techniques can also be carried out on the thin sections or on sediment blocks. Micromorphological samples were collected from major stratigraphic units in DCM and DCE. Sediment blocks were extracted by scoring the area to be sampled with a knife and then covering it with plaster of Paris bandages to preserve the structural integrity^72^. Sample blocks were variable in size, but were typically around 20 cm tall by 10 cm wide and extended 10 cm deep into the stratigraphic profile. Blocks were shipped to the Geoarchaeology Laboratory at the Centre for Archaeological Science, University of Wollongong, where they were oven dried at 40 °C. The dried blocks were impregnated with Dalchem crystic polyester resin diluted with styrene (ratio of 7:4) and catalysed with methyl ethyl ketone peroxide (12.5 ml per litre of resin/styrene mixture). After curing, the samples were oven dried overnight at 50 °C and trimmed to 50 × 75 mm ‘wafers’ that were shipped to Spectrum Petrographics (Vancouver, WA, USA) for final thin-section manufacture; some thin sections were made by J. Abrantes in the School of Earth, Atmospheric and Life Sciences at the University of Wollongong. Thin sections were first scanned on a flatbed scanner at 2400 dpi, both in reflection mode and without the flatbed cover^73^ to provide an overview of the general composition. Thin-section examination was carried out with stereoscopic and petrographic microscopes at magnifications ranging from 8× to 200× under plane- and cross-polarised light. Thin-section terminology follows that of Stoops^74^. For the SEM-EDS analysis, uncoated thin sections were analysed using a bench-top Phenom XL scanning electron microscope with a CeB_6_ source and a built-in energy-dispersive X-ray spectrometer (EDS) housed at the School of Earth and Environmental Sciences, University of Wollongong. Back-scattered electron (BSE) images/secondary electron (SE) images were collected at 5/10/15 kV at low vacuum (60 Pa)/medium vacuum (10 Pa)/high vacuum (1 Pa). Semi-quantitative EDS maps were collected at 5 kV at low vacuum. These data were processed using PhenomProSuite elemental identification software.

## Supplementary information

Morley et al Supplementary Information
