# Author Correction: Hominin and animal activities in the microstratigraphic record from Denisova Cave (Altai Mountains, Russia)

**DOI:** 10.1038/s41598-021-03251-6

**Published:** 2022-01-24

**Authors:** Mike W. Morley, Paul Goldberg, Vladimir A. Uliyanov, Maxim B. Kozlikin, Michael V. Shunkov, Anatoly P. Derevianko, Zenobia Jacobs, Richard G. Roberts

**Affiliations:** 1 Centre for Archaeological Science, School of Earth, Atmospheric and Life Sciences, University of Wollongong, Wollongong, New South Wales 2522 Australia; 2 Archaeology, College of Humanities and Social Sciences, Flinders University, Adelaide, South Australia 5042 Australia; 3 Institut für Naturwissenschaftliche Archäologie, Eberhard-Karls-Universität Tübingen, Rümelinstrasse 23, 72070 Tübingen, Germany; 4 Institute of Archaeology and Ethnography, Russian Academy of Sciences, Siberian Branch, Novosibirsk, 630090 Russia; 5 Lomonosov Moscow State University, Moscow, 119991 Russia; 6 Novosibirsk State University, Novosibirsk, 630090 Russia; 7 Australian Research Council (ARC) Centre of Excellence for Australian Biodiversity and Heritage, University of Wollongong, Wollongong, New South Wales 2522 Australia

Correction to: *Scientific Reports* 10.1038/s41598-019-49930-3, published online 26 September 2019

The original version of this Article contained errors in Figure 3, in panels (a) and (b), where the ‘Initial Upper Palaeolithic’ was incorrectly given as ‘Initial Middle Palaeolithic’.

In addition, in panel (a), the break of the middle MP and the IUP between layers 12.1 and 11.4 was incorrectly placed between layers 14 and 12.3.

The original Figure 3 and accompanying legend appear below.

**Figure 3:**
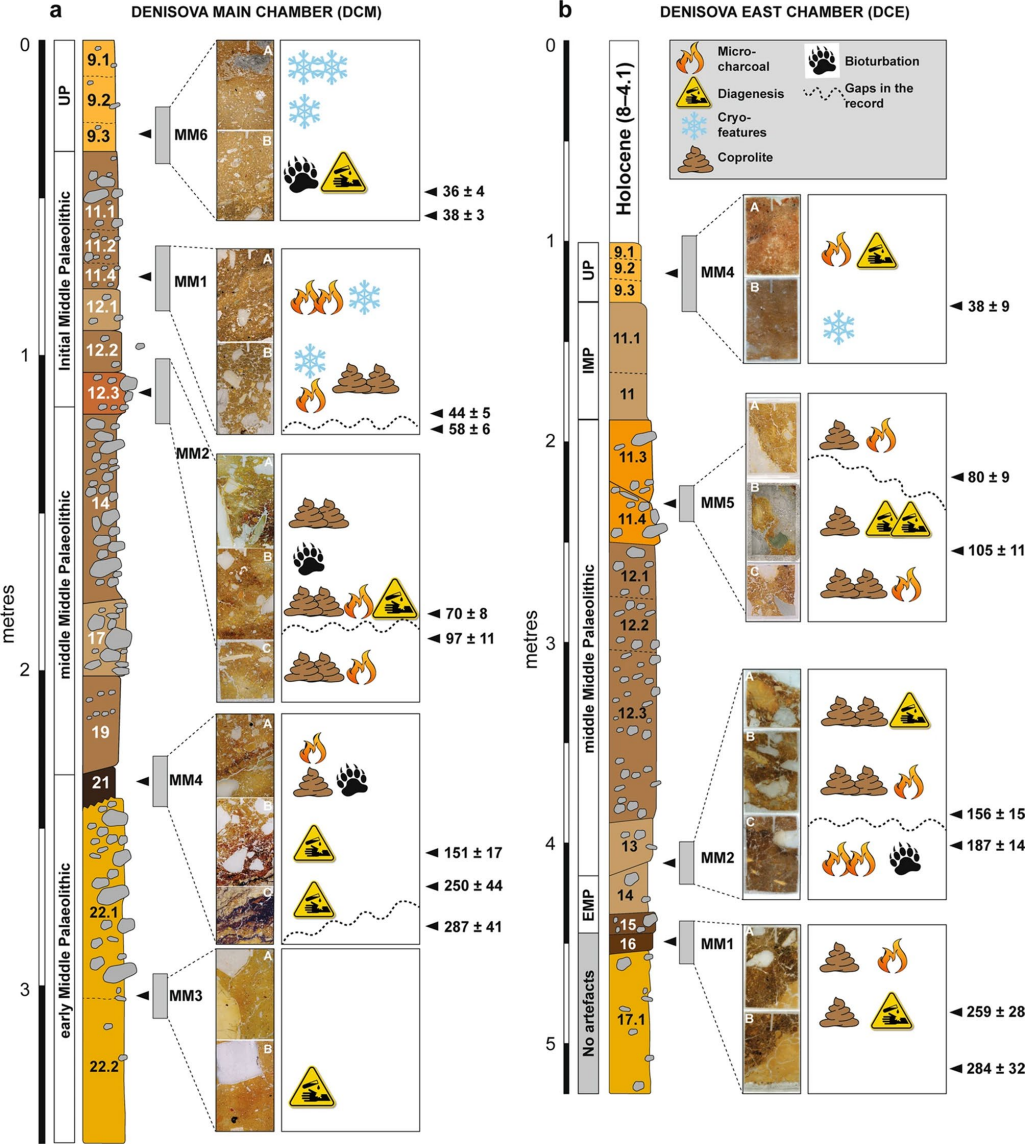
Summary stratigraphic logs of the sequences exposed in (**a**) DCM and (**b**) DCE, showing the locations of the micromorphological samples and key microstratigraphic features. To the right of each log, optical ages (in ka, with uncertainties at 95.4% probability) are shown for the major boundaries between lithological units in the thin sections, together with the associated archaeological phases (from ref.^9^).

The original Article has been corrected.

