# Supplementary material for: Hominin and animal activities in the microstratigraphic record from Denisova Cave (Altai Mountains, Russia)
Source: Sci Rep. 2019 Sep 26;9:13785. doi: 10.1038/s41598-019-49930-3 (PMC6763451; doi:10.1038/s41598-019-49930-3)
Supplement: Supplementary file 1 — Morley et al Supplementary Information [file 41598_2019_49930_MOESM1_ESM.pdf]

Hominin and animal activities in the microstratigraphic record from Denisova Cave (Altai Mountains, Russia)

Mike W. Morley, Paul Goldberg, Vladimir A. Uliyanov, Maxim B. Kozlikin, Michael V. Shunkov, Anatoly P. Derevianko, Zenobia Jacobs & Richard G. Roberts

Supplementary information

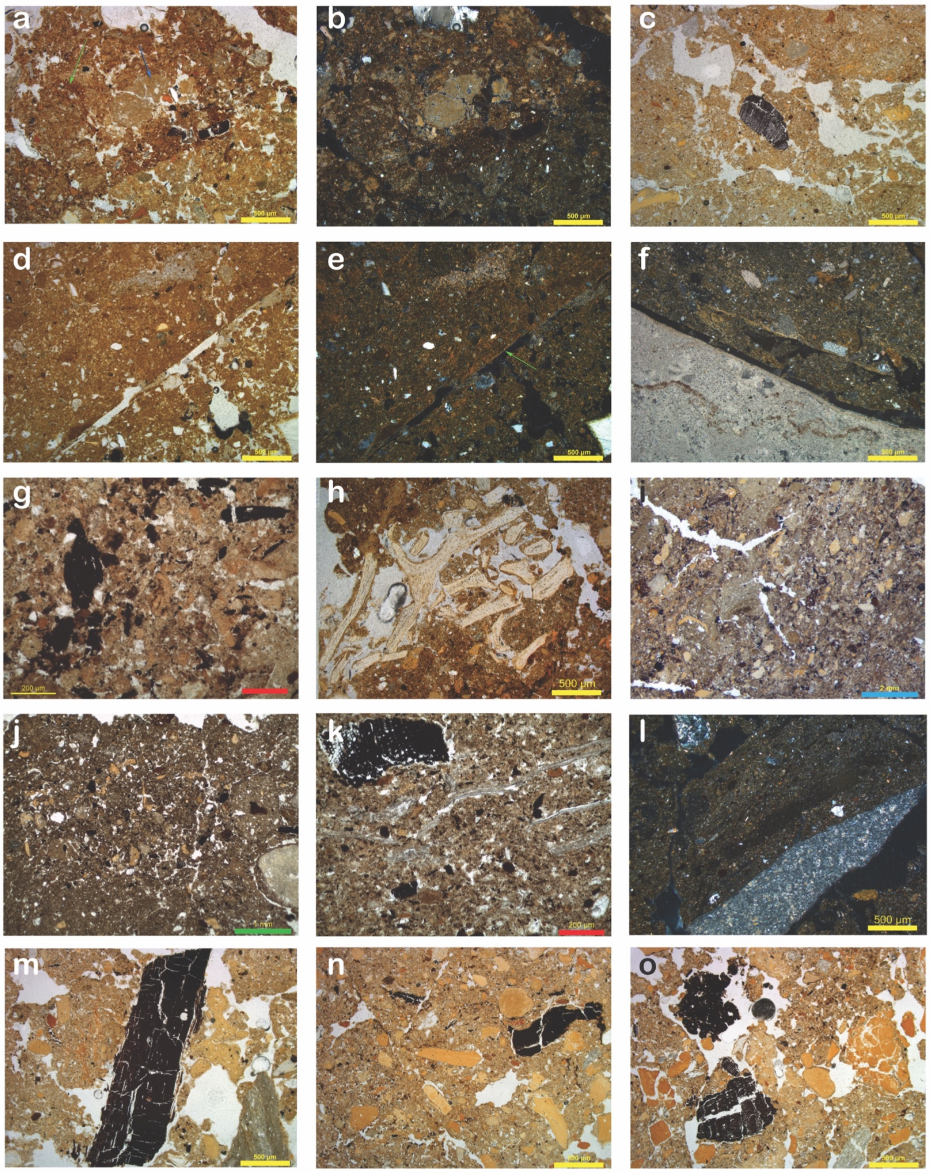

Figure S1. Photomicrographs of key features of the Denisova Cave microstratigraphy mentioned in the main text: **a**, **b**, DCM-MM4A, layer 20. Interface between pale brown lower unit and dark brown band running through centre of the thin section. The colouration of the dark band is due to charcoal fragments and finely divided charcoal powder interspersed throughout the matrix. Angled interface and shear zones seen throughout are consistent with fracturing and faulting of the sediments, possibly linked with plastic deformation (see panels **c–f**). Bioturbation is also evident, with infilled burrows visible (note burrow in dark band indicated by green arrow, and weathered grain shown by blue arrow) (ppl and xpl, respectively); **c**, DCM-MM4A, layer 20. Rounded charcoal fragment with preserved cellular structure in upper area of thin section (ppl); **d**, **e**, DCM-MM3B, layer 22.1. Shear plane with alignment of clay along void (green arrow in panel **i**), and limestone fragment in advanced state of dissolution (ppl and xpl, respectively); **f**, DCM-MM3A, layer 22.1. Shear plane along the margins of a limestone clast, showing granostriated b-fabric (xpl); **g**, DCE-MM1B, layers 17–15. Humified organic material and fine charcoal pieces in a compact matrix (ppl); **h**, DCE-MM1A, layers 17–15. Fractured and crushed bone fragments, possibly related to trampling (ppl); **i**, DCE-MM1A, layers 17–15. Humified organics and charcoal fragments in a densely compressed sediment matrix;

j, DCE-MM2C, layers 14 and 13. Very fine bone fragments in a fine silty matrix, within the dark central band. This layer may be a bioturbated occupation horizon (ppl); **k**, DCE-MM2C, layers 14 and 13. Charcoal fragment with preserved plant cellular structure and bedded phytoliths (ppl); **l**, DCE-MM2C, layers 14 and 13. Angular rock fragment, probably a piece of micro-debitage from the manufacture of a stone artefact (xpl); **m**, DCE-MM5C, layer 11.4. Large charcoal fragment (ppl); **n**, DCE-MM5C, layer 11.4. Heterogeneous mix of coarse material, with fine bedded charcoal and deformed charcoal fragment, indicative of downslope movement and/or plastic deformation of the sediments (ppl); **o**, DCE-MM5A, layers 11.4 and 11.3. Poorly sorted coarse components in a fine silty matrix. The coarse fraction includes high frequencies of rounded clay aggregates, many of which are cracked, presumably through desiccation. Some large pieces of charcoal are also present, some of which occur in large void spaces and are broken, possibly due to bioturbation (ppl). Scale bars: red, 200 µm; yellow, 500 µm; green, 1 mm; blue, 2 mm.

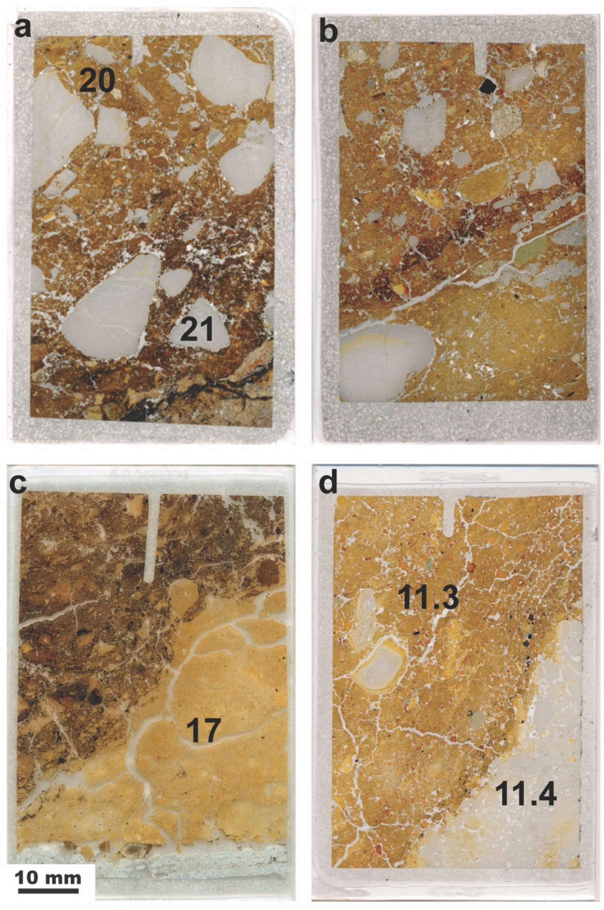

Figure S2. Thin section flatbed scans. **a**, thin section DCM-MM4B showing interface between layers 21 and 20 at the base of the section. Note the finely bedded humified organics of layer 21 in the lower right-hand corner, indicating a humid environment and the accumulation of organic material on the irregular surface of the cave floor; **b**, thin section DCM-MM4A, with dark band running through layer 20 caused by the presence of fine charcoal powder interspersed throughout the sediment matrix; **c**, thin section DCE-MM1B showing the sub-horizontally truncated upper surface of layer 17, overlain by fine-grained sediments rich in micro-charcoal, the oldest combustion bi-products yet recorded at Denisova Cave (259 ± 28 ka); **d**, thin section DCE-MM5A, showing the angled interfaces between layers 11.4 and 11.3 caused by slumping and compression of older layers relating to the hour-glass feature and basal karstic tube. A Neanderthal toe phalanx (Denisova 5) was recovered from layer 11.4; micro-charcoal is recorded in this and the overlying layer, with larger charcoal fragments evident just above the interface.

Table S1. Summary macro- and micro-descriptions of the main Pleistocene layers in the Main and East Chambers of Denisova Cave. Start (*) and end (†) ages and archaeological phases from ref. 9, with the age uncertainties expressed at the 95.4% confidence interval

Layer	Macro- and micro-description	Environment	Start / end age (ka) and archaeological phase
**Denisova Main Chamber (DCM)**			
**22**	Yellowish-brown, clay silt with gravel inclusions, speleothem frags and fresh bones. Compound aggregate 'rip-up clasts' are common, with internal stratification consistent with original deposition under aqueous conditions. Speckled and granostriated b-fabric, with clay orientation along micro-fractures, shear zones and faults. Calcite etching and localised manganese formation. High porosity towards upper surface. The upper surface is truncated by low-energy water drainage.	Reworked phreatic sediments, with signs of instability following erosion. Weak diagenesis and bioturbation.	287 ± 41†<br><br>early Middle Palaeolithic
**21**	Very finely stratified minerogenic and organic laminae. Dark anastomosing organic bands interstratified with yellowish brown and pale grey silts. Locally rich in siliceous plant remains, including phytoliths and horizontally emplaced vegetal fibres. Strong diagenetic alterations, including phosphatic zones, limestone weathering, and presence of precipitated apatite. Small bone fragments and fine charcoal. Coprolites present.	Damp cave environment, irregular cave floor surface. Diagenetic processes catalysed by the presence of water.	250 ± 44*<br><br>early Middle Palaeolithic
**20**	Dark, porous, and crumbly sandy silts, containing frequent, silt-sized charcoal fragments and finely divided charcoal powder dispersed throughout the matrix. Phosphate nodules and phosphatised speleothem/limestone fragments. At the interface with layer 19 is a dark brown organic band containing fine charcoal fragments. Micro-fracturing and faulting is evident along bedding planes, and the parallel arrangement of mica indicates slippage and shear zones.	Unstable cave floor environment with anthropogenic inputs.	170 ± 19†<br><br>early Middle Palaeolithic
**19**	Yellowish-brown, poorly sorted clay silt with frequent coarse inclusions, such as fine gravel of various lithologies (e.g., schist, marble and siltstone). Frequent large bones, coprolite fragments, degraded limestone, and bone.	Debris flow material intermixed with evidence for animal occupation.	151 ± 17*<br><br>middle Middle Palaeolithic
**17**	Coarse, clast-supported gravels in a silty sand matrix. Not sampled for micromorphology owing to the coarse composition.	Talus and debris flow.	128 ± 13†<br><br>middle Middle Palaeolithic
**14**	Moderately compact, friable silt with clay. Poorly sorted with massive angular gravels. Towards upper horizon becomes coarser, gritty silt with very frequent fine to medium limestone clasts. Contains elongate bone splinters and bone fragments with signs of etching. Carbonate clasts (limestone and speleothem) and calcite sand are in the process of dissolution.	Variable environments represented, with pulses of debris flow punctuated by animal activity and presence of water.	112 ± 12*<br><br>97 ± 11†<br><br>middle Middle Palaeolithic

**12.3/ 12.2/ 12.1**	Layer 12.3 forms a distinctive reddish-brown band, consisting of a heterogeneous and poorly sorted mix of coarse inclusions. Overlain by layer 12.2, pale yellowish-brown with frequent coprolites. Elongate limestone clasts are vertically oriented within the matrix. Layer 12.1 is a pale, yellowish-brown silt with angular coprolites, possibly suggesting fresh breaks, and weak chemical diagenesis. Incipient platy structures are present.	Animal activity and a move to cooler and possibly more humid conditions.	70 ± 8*<br><br>58 ± 6†<br><br>middle Middle Palaeolithic
**11.4**	Gravel-rich layer with a compact silty clay matrix and inclusions of sand-sized charcoal fragments and flecks. Limestone gravel clasts are fresh, with no obvious signs of diagenetic alteration. Sediment structure is variable, with poorly developed platy regions and regions where large (~1 mm) polygonal blocks are present. Carbonates are well preserved, with only occasional signs of phosphatisation.	Generally dry environment with evidence for hominin activity.	44 ± 5*<br><br>Initial Upper Palaeolithic
**11.2**	Silty clay with a marked increase in charcoal and reduction in gravel. Charcoal, where present, is finer than in layer 11.4, and charcoal powder provides a dark colour. Coprolites and bone fragments are generally small and rounded. A platy structure and rotational structures are present and some limestone clasts have silt cappings. Some well-preserved, large (~1 mm) charcoal pieces retain the original plant cellular structure. A distinct yellow band is present within layer 11.2 containing frequent coprolites and less charcoal. Charcoal powder increases towards the upper boundary, providing a darker colouration, and the platy structure is less developed and the intra-pedal blocks are smaller and more rounded.	Pulses of hominin and other animal activity suggested, occurring against a cold climate.	Initial Upper Palaeolithic
**11.1**	Dark, non-calcareous fine silt, frequently manganese stained. Coprolite fragments are common and variably rounded. Limestone clasts show signs of phosphatisation and bone fragments are often poorly preserved. The silt that forms the matrix has a fluffy texture and is free of impurities and inclusions, with the exception of occasional charred and humified angular plant fragments.	Carnivore activity, humid conditions and introduction of plant matter.	38 ± 3†<br><br>Initial Upper Palaeolithic
**9.3/ 9.2**	Fine grained silts and silty clays with poorly sorted coarse components. Sediment structure here is dominated by vughs and channel voids. The matrix is composed of silt aggregates about 100–150 μm in size, providing a 'fluffy' appearance. Layer 9.2 contains fewer voids and has a platy structure that is finely expressed. The matrix here is finer, bioturbation is present, with infilled burrows and voids. Large aggregate grains are rich in plant remains and biogenic silica, probably phytoliths.	Possibly aeolian input to the cave during a cool climate regime. Fine grained sediments blown to the site from the valley flanks.	36 ± 4*<br><br>21 ± 8†<br><br>Upper Palaeolithic

Denisova East Chamber (DCE)			
**17**	Very fine, pale yellow silty clay, coarsening upwards. The upper interface is truncated at an angle of 45°. Shear zones and signs of slippage are evident towards the truncated surface, suggesting erosion and plastic deformation of the upper surface. Occasional rodent/bird bones and rounded aggregates of pure clay are also present. Post-depositional features include gleying, deferrification and fragments of phosphatic crust and/or coprolite.	Reworked phreatic sediments, horizontally truncated and destabilised (similar to layer 22 in DCM).	284 ± 32†<br><br>No artefacts
**16**	Dark brown, clay silts with frequent humified and iron-stained organics and micro-charcoal. Plant remains include large (about 1–2 mm) pieces of iron-stained wood and other fibrous material that are severely diagenetically transformed. Frequent rip-up clasts consist of reworked yellow layer 17 sediments. Rotational features are present locally where the matrix has been organised into rounded clumps of material. Lower porosity in the upper part is consistent with compression, possibly by trampling.	Environment conducive to the preservation of organic material, coincident with the presence of hominins and the use of fire.	259 ± 28*<br><br>238 ± 20†<br><br>No artefacts
**15**	Pale brown, densely compact silty clays. Trampling is evident in the form of crushed and fractured bone and charcoal fragments. There are also zones of pale and pure micaceous clay, banded within the matrix, which may represent deferruginised zones.	Hominin activity in a generally humid environment.	203 ± 14*<br><br>197 ± 12†<br><br>early Middle Palaeolithic
**14**	Pale brown silts with poorly sorted coarse inclusions. Generally porous and loose with signs of bioturbation. Dendritic manganese veins are present but, in general, there are only minor signs of diagenesis. The poorly sorted coarse material includes small fragments (~100 μm) of well-preserved bone and small charcoal pieces. An abrupt contact exists with an overlying band comprising a fine, well sorted, densely compact silt (possibly aeolian). Coarse components include well-preserved, rounded clay aggregates, but otherwise the sediment is clean and homogeneous with only occasional inclusions (e.g., small bone fragments).	Animal and hominin activity with an influx of windblown silts (reworked?). Possible redox environment related to pulses of water?	193 ± 12*<br><br>187 ± 14†<br><br>early Middle Palaeolithic
**13**	Dark brown clay silts. Marked increase in charcoal and humified organic matter. A large bone fragment shows signs of step fracturing that may be consistent with trampling. Very frequent coprolite fragments, some of the larger pieces are broken, possibly during surface desiccation. There are also strong signs of bioturbation in the form of planar, channel voids, and vughs. In the upper region, the sediments contain poorly sorted mixtures of large and small coprolite fragments, limestone clasts (some of which are subject to chemical dissolution), small- to medium-size bone fragments, and rock fragments of assorted lithologies.	Hominin and other animal activity, presumably pulsed as extremely unlikely to cohabitate.	156 ± 15*<br><br>146 ± 11†<br><br>middle Middle Palaeolithic

**11.4**	Pale yellow silty clays with a heterogeneous mix of poorly sorted coarse components. Fine charcoal is dispersed throughout the matrix, and well-preserved bone fragments and coprolites are common. Phytoliths are also common, but smaller in size than those recorded in DCM. Phosphatisation of limestone grains has occurred, and dark coprolite fragments are present. The sediments are generally low in calcite, but localised calcitic zones occur, possibly the result of decomposed limestone. The interface between layers 11.4 and 11.3 is aligned at an angle of ~45°.	Heterogeneous deposit, evidence of hominin and other animal occupation. Destabilisation and erosion of cave floor relating to slumping and compression.	120 ± 11* (layer 12.1)<br><br>105 ± 11†<br><br>middle Middle Palaeolithic
**11.3**	Darker brown silty clays with frequent charcoal pieces. Some charcoal fragments are compressed and smeared, suggesting transport before undergoing plastic deformation and slipping. Coarse fraction is dominated by rounded (and sometimes cracked) clay aggregates. Limestone clasts have well-developed phosphate crusts. Coprolites are much darker orange, often rectangular in shape, and have a massive internal composition with fewer inclusions. Phytoliths are concentrated in localised areas. A compound aggregate is also present, comprising what appears to be coprolites in a heterogeneous matrix of silt, clay and fine sand.	Possible change in primary user of the cave.	80 ± 9*<br><br>70 ± 8†<br><br>middle Middle Palaeolithic
**11.1**	Pale, fine silt with frequent coarse gravel. The matrix varies from platy to rotational with evidence for granostriated b-fabric surrounding several rock fragments. Frequent inclusions of pale yellowish-brown coprolites and bone fragments. Limestone clasts are often severely phosphatised, with the surrounding phosphatised rind isotropic in cross-polarised light. Fine charcoal flecks and fragments are present, dispersed throughout the matrix. An angled sharp interface may be the result of burrowing of the surface of the lower unit. The darker colouration in the upper part is due to an increase in charcoal and humified organic inclusions.	Variable conditions, with pulses of bedrock attrition. Hominin and other animal activity.	49 ± 6*<br><br>38 ± 9†<br><br>Initial Upper Palaeolithic
**9.3/ 9.2/ 9.1**	Pale brown clay silt with occasional charcoal fragments and flecks. Weakly developed platy structure and aggregate grains with silt cappings. Organic inclusions increase in frequency towards the upper horizon, some of which shows signs of haematite staining.	Cooler conditions and the presence of animal activity.	after 38 ± 9<br><br>Upper Palaeolithic
